# Supplementary material for: Metatranscriptomic investigation of single Ixodes pacificus ticks reveals diverse microbes, viruses, and novel mRNA-like endogenous viral elements
Source: mSystems. 2024 May 14;9(6):e00321-24. doi: 10.1128/msystems.00321-24 (PMC11237458; doi:10.1128/msystems.00321-24)
Supplement: Supplemental Figures — Figures S1-S8. [file msystems.00321-24-s0001.pdf]

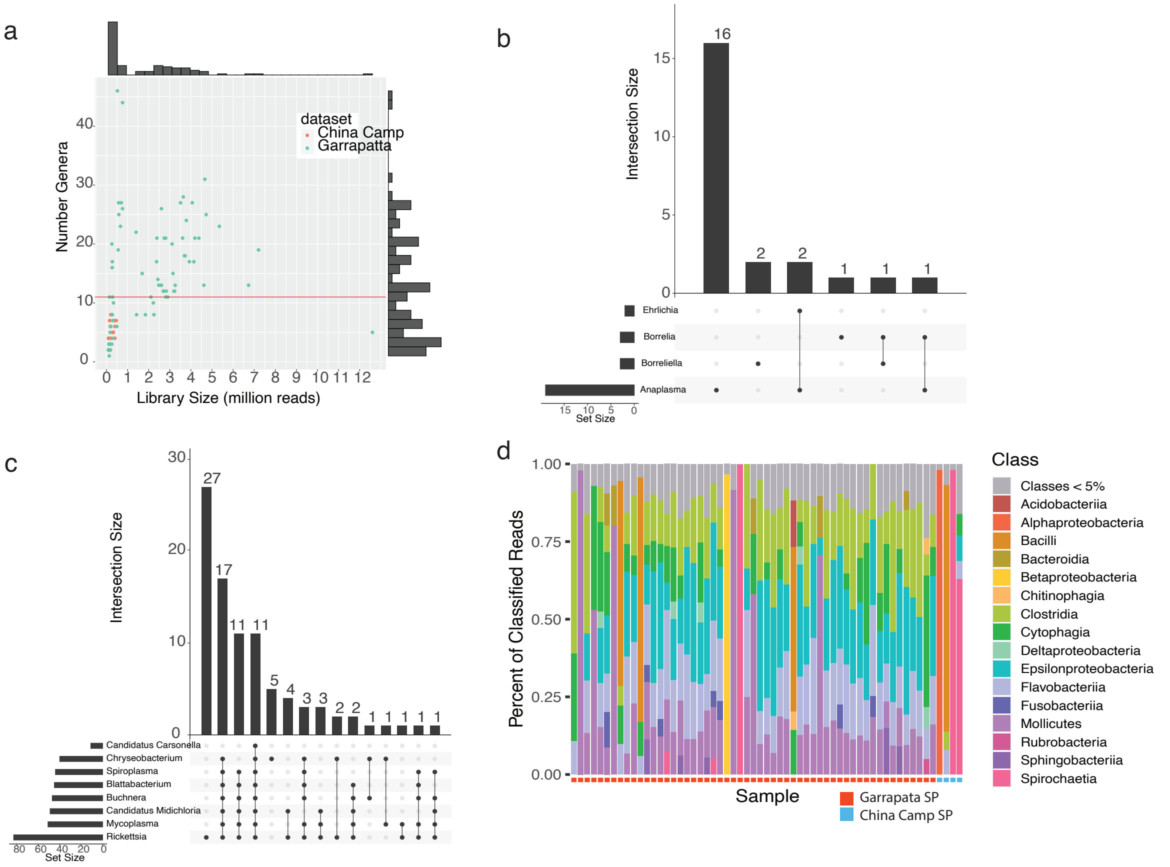

Figure S1: Pathogens and commensals of *I. pacificus* a) Scatterplot displaying number of nonhost reads and number bacterial genera detected per sample. Horizontal line shows median number of genera across all samples. Histograms on each axisrepresentdistribution of axis values. b)Upset plot displaying number of coinfections of known bacterial pathogens. Numbers represent number of samples with the included set of genera. c) Upset plot displaying number of coinfections of newly identified endosymbionts. d) Strip plot displaying the propotion of reads assigned to each bacterial class by sample.

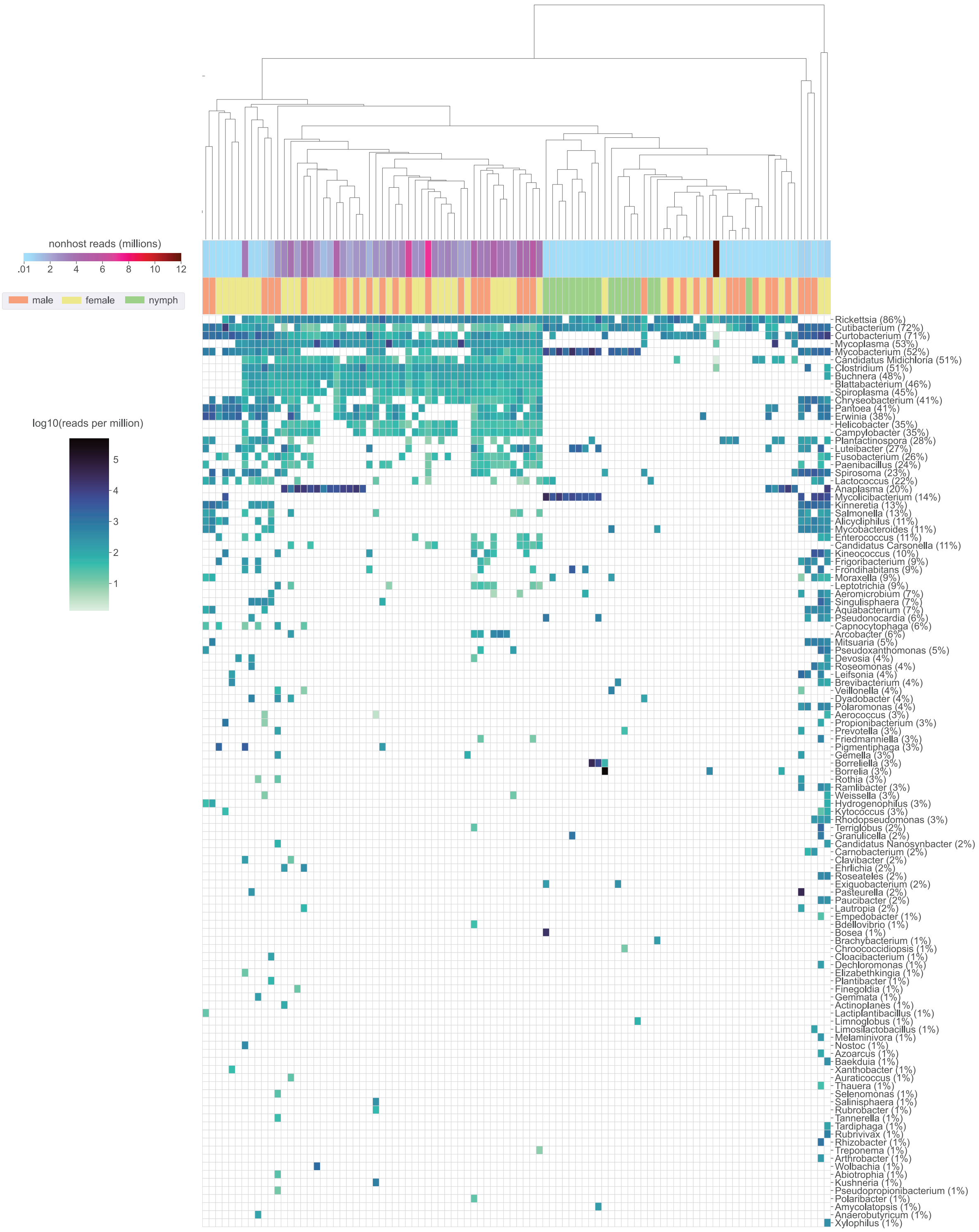

Figure S2: Bacterial genera of *I. pacificus* Heatmap showing reads per million (rpm) of bacterial genera as classified by kraken2. Rows are ordered in decreasing prevalence (shown next to genus name) and columns are heirarchically clustered by euclidean distance.

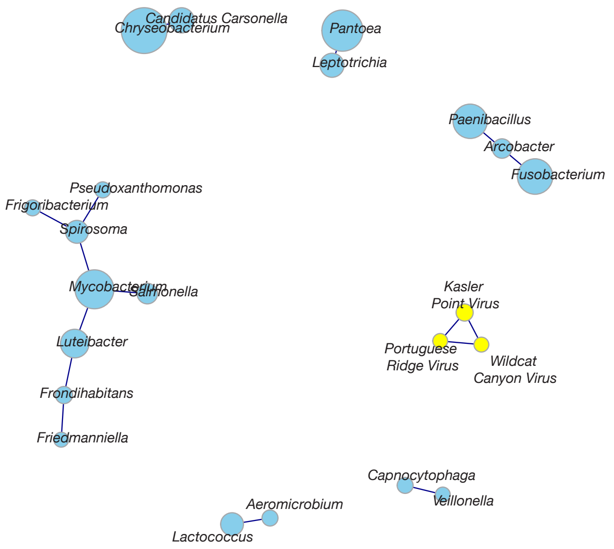

Figure S3: Co-occurrence of bacterial and viral taxa Network representation of significant co-occurring relationships amongst all identified viruses and bacterial genera. Sizes of nodes are scaled to the prevalence in the dataset. All edges represent a positive co-occurence of alpha value greater than or equal to 5 with a p-value less than or equal to 0.05

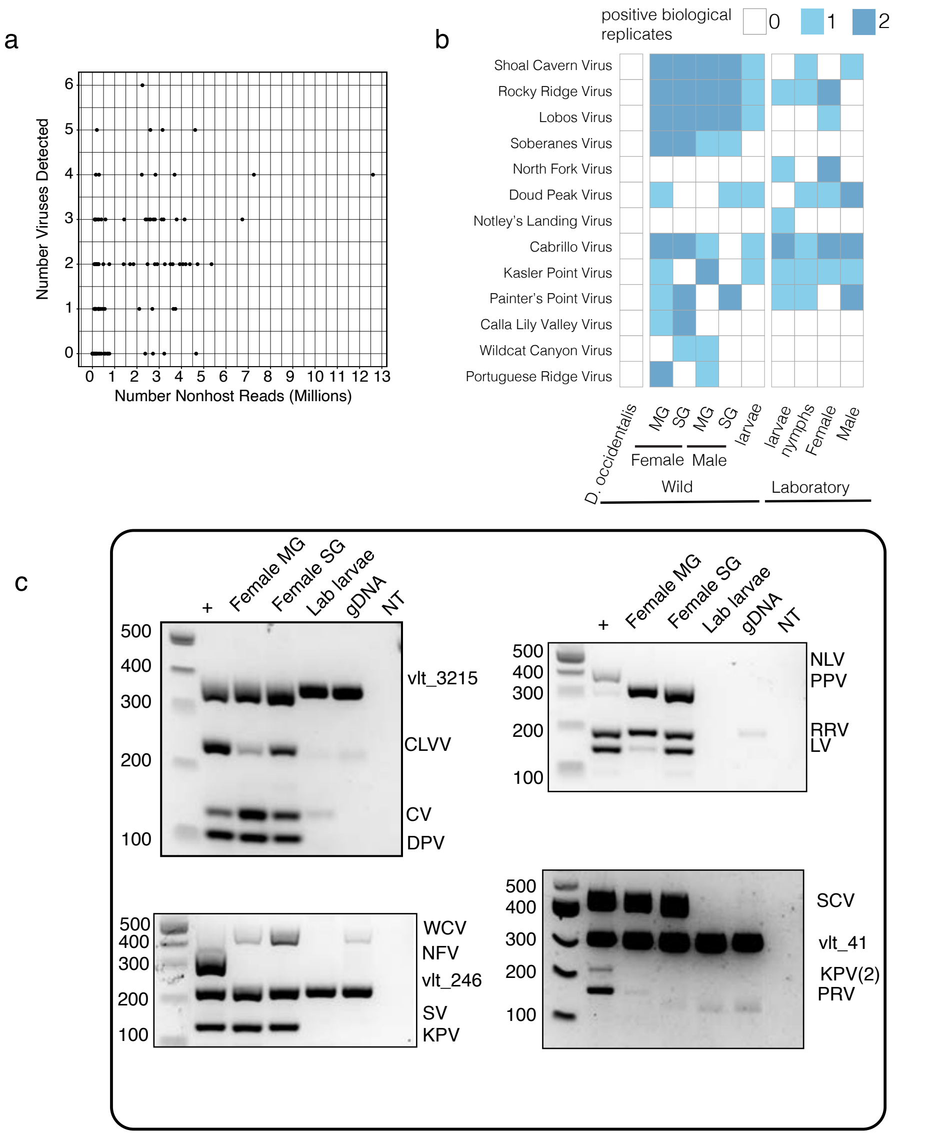

Figure S4: Discovered viruses in *I. pacificus* a) Scatterplot of nonhost library size and number of viruses detected per sample. b) summary of PCR results for tick tissues and life stages. Results for adults are shown separately by sex and the number of biological replicates (individual pools of ticks or tissues) is indicated by color c) Representative gel image of viruses tested in midguts and salivary glands and lab-reared larvae. Virus abbreviations are aligned to their expected band size. Bands beginning with "vlt" represent virus-like transcripts (see Figure 4).

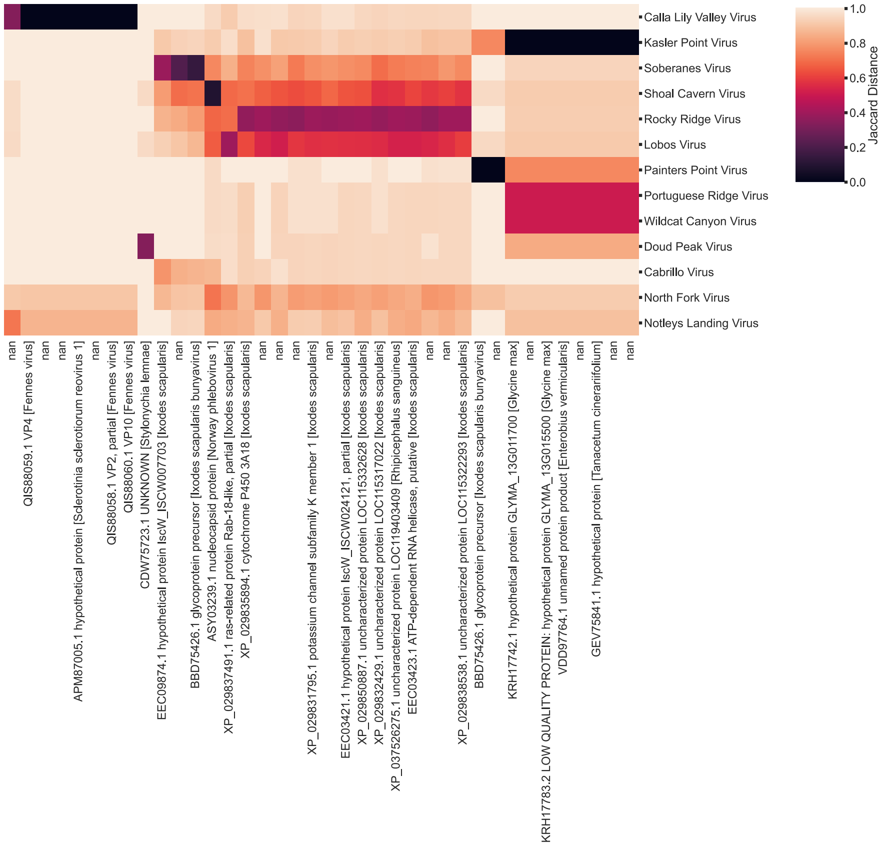

Figure S5: Identification of additional viral genomic segments Heatmap of Jaccard distance of rdrp-containing contigs (rows) and other contigs in the dataset (columns). Columns are annotated with the closest blastx hit or nan if no hits were found.

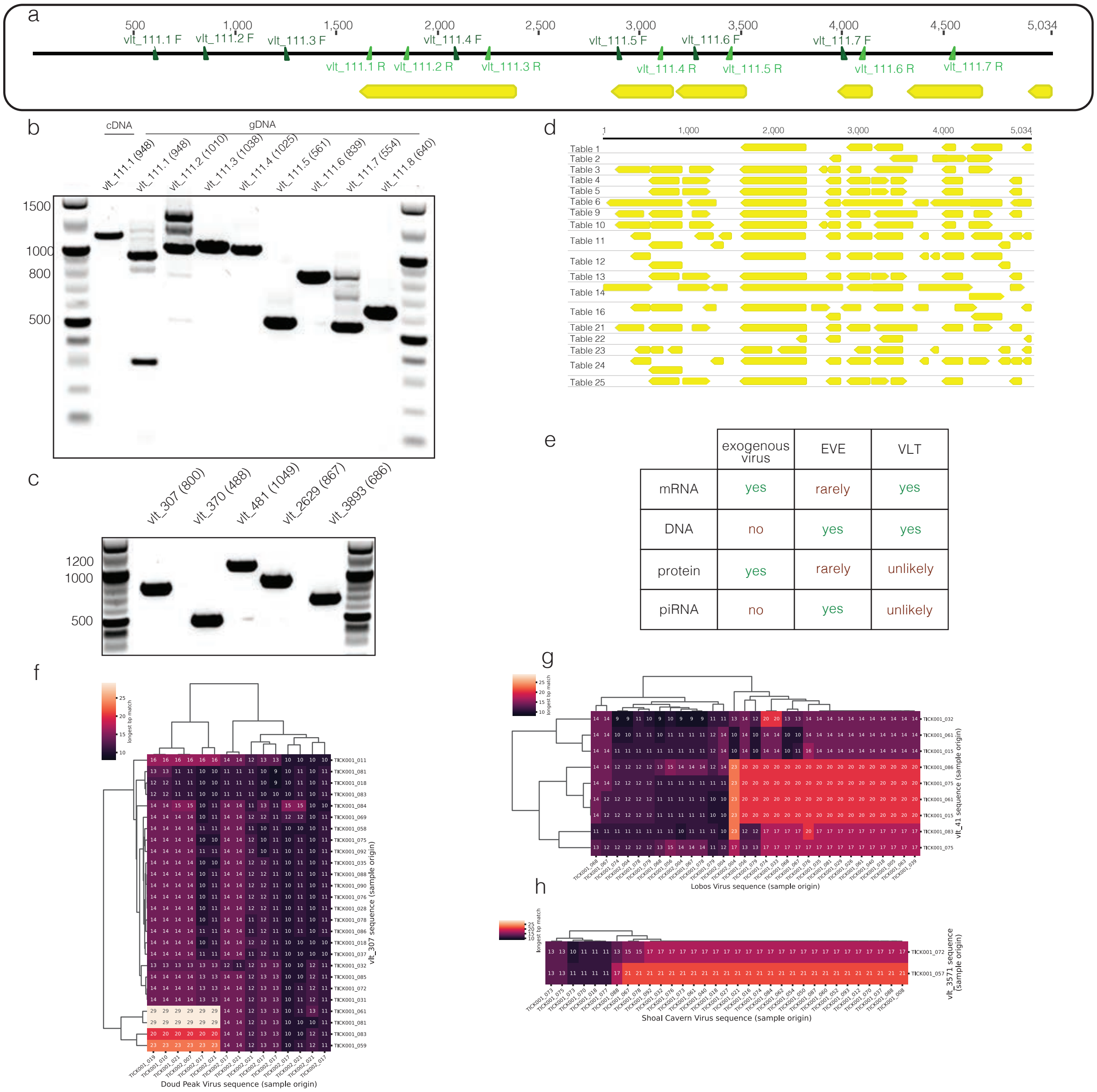

Figure S6: Confirmation of VLT sequence and presence in DNA a)Visual representation of c111 identified from RNA seq, including predicted open reading frames (yellow) and primer pairs b) PCR reactions amplifying the regions indicated in a, nucleic acid type indicated on lane. Expected band size in base pairs indicated in parentheses. c) PCR reactions from an additional 5 virus-like sequences amplified from gDNA, expected band size indicated in parentheses. d) Open reading frames predicted for c111 using alternative codon tables e) Table summarizing expected pattern of exogenous viruses, endogenous viral elements (EVEs) and observed pattern of VLTs f) Clustermap displaying the length of the longest perfectly matching sequence between each sequence assigned to the Doud Peak Virus cluster and each sequence assigned to the vlt_307 cluster. Rows and columns are labeled with the tick sample from which the sequence originated g-h) As in a but for Lobos Virus:vlt_41 and Shoal Cavern Virus:vlt_3571 respectively

a

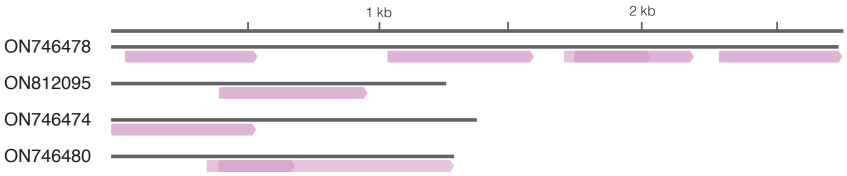

b

VLT	Sequence	Organism	Description	Percent Identity	Alignment Length (bp)	Bitscore	evalue
vlt_2742	AC192419.4	Ixodes scapularis	Ixodes scapularis, clone XX-43A1, complete sequence	98.35	121	210	4.17E-52
vlt_481	XM_042294477	Ixodes scapularis	PREDICTED: Ixodes scapularis zinc finger protein 14-like (LOC115327220), transcript variant X2, mRNA	90.65	139	192	1.94E-46
vlt_2636	AC192419.4	Ixodes scapularis	Ixodes scapularis, clone XX-43A1, complete sequence	96.23	106	174	3.03E-41
vlt_3893	XM_050180920	Dermacentor andersoni	PREDICTED: Dermacentor andersoni alpha-ketoglutarate-dependent dioxygenase alkB homolog 4-like (LOC126533730), mRNA	87.18	39	45.5	0.033
vlt_370	XM_040219523	Ixodes scapularis	PREDICTED: Ixodes scapularis piggyBac transposable element-derived protein 3-like (LOC120847660), mRNA	90.08	131	178	4.54E-42
vlt_20	XM_040219523	Ixodes scapularis	PREDICTED: Ixodes scapularis piggyBac transposable element-derived protein 3-like (LOC120847660), mRNA	91.61	155	222	9.67E-55
vlt_1472	XM_029970693	Ixodes scapularis	PREDICTED: Ixodes scapularis uncharacterized LOC115311839 (LOC115311839), mRNA	75.00	68	47.3	0.013
vlt_27	XR_003919753.	Ixodes scapularis	PREDICTED: Ixodes scapularis uncharacterized LOC115327269 (LOC115327269), transcript variant X1, ncRNA	73.63	91	57.2	2.23E-05
vlt_1071	XM_042288860	Ixodes scapularis	PREDICTED: Ixodes scapularis uncharacterized LOC8039455 (LOC8039455), transcript variant X2, mRNA	75.31	239	166	2.09E-38
vlt_307	AC192742.6	Ixodes scapularis	Ixodes scapularis, clone XX-48A1, complete sequence	75.72	313	229	3.08E-57
vlt_1557	XM_029974126	Ixodes scapularis	PREDICTED: Ixodes scapularis mitochondrial folate transporter/carrier (LOC8041517), mRNA	99.33	149	265	2.60E-68
vlt_111	XM_040499670	Ixodes scapularis	PREDICTED: Ixodes scapularis uncharacterized LOC120837521 (LOC120837521), transcript variant X1, mRNA	88.30	342	437	4.46E-120
vlt_246	OP068155.1	Ixodes ricinus	Ixodes ricinus isolate Ixodes ricinus/Moscow/264/2020 endogenous virus Jingmen tick virus RNA-dependent RNA polymerase gene, partial cds	75.19	2229	1517	0

Figure S7: VLTs in other datasets a) The ORF structure of four sequences as reported by Ni et al. b)A table of blast results for the 25 representative VLT sequences

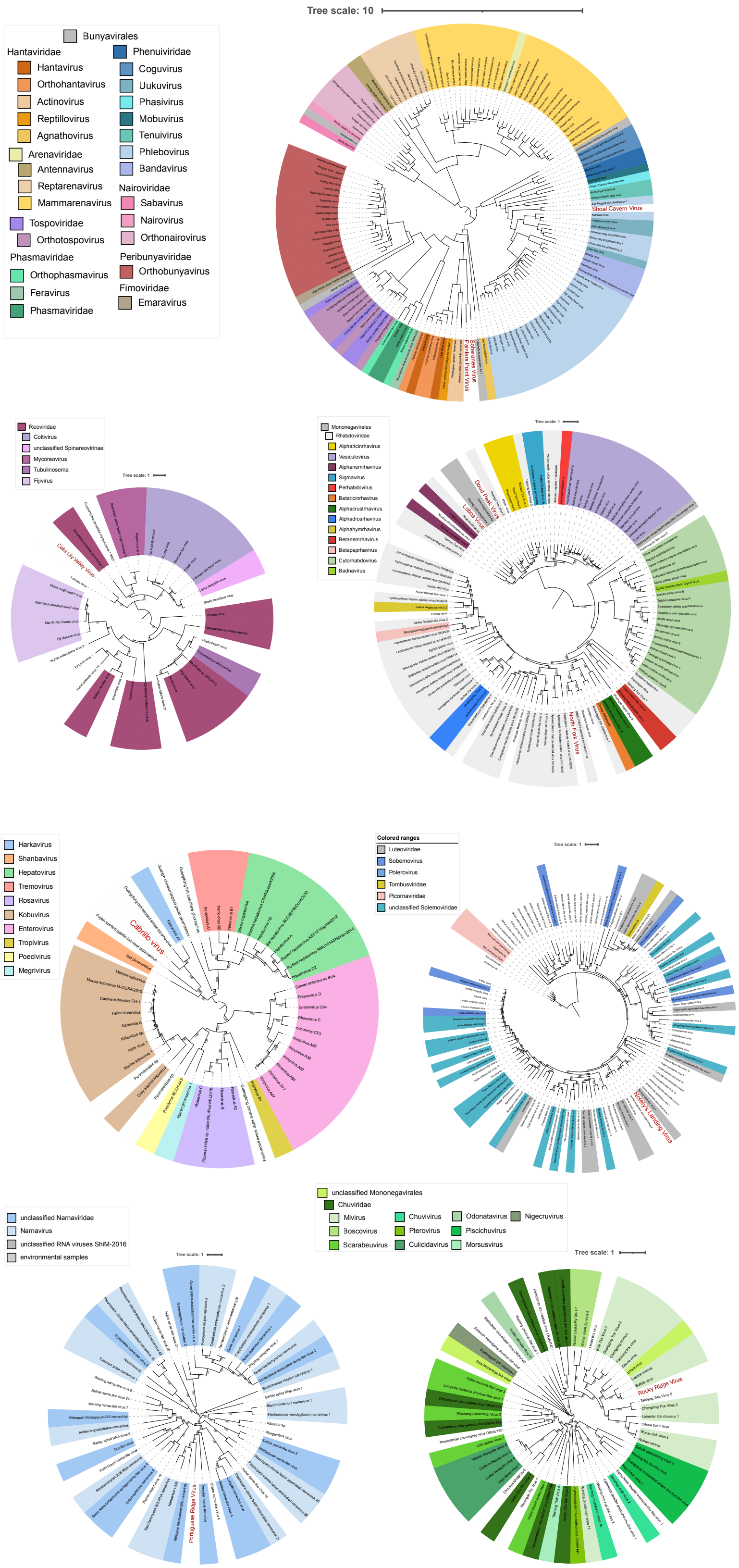

Figure S8: Phylogenetic placement of novel viruses Legends display genus for each leaf, or higher level classification if genus unclassified (family or order). Leaves with no color had no taxonomic classification. Branch points display bootstrap support as a percentage
