# Supplementary material for: Metatranscriptomic investigation of single Ixodes pacificus ticks reveals diverse microbes, viruses, and novel mRNA-like endogenous viral elements
Source: mSystems. 2024 May 14;9(6):e00321-24. doi: 10.1128/msystems.00321-24 (PMC11237458; doi:10.1128/msystems.00321-24)
Supplement: Supplemental Note — Descriptions of viral genome assemblies. [file msystems.00321-24-s0002.docx]

**Supplementary Note**

As described in Methods, we followed NCBI/ICTV demarcation methodology for taxonomically annotating our viral genomes based on RNA-dependent RNA polymerase (RdRp) sequence similarity to known viral taxa. For each distinct genome sequence, we recovered (Table S1), we visualized RdRp sequence similarities of the top 100 BLAST hits using phylogenetic trees in iTOL (Figure S8). The following descriptions summarize what is known about the the closest relatives of our viral genomes, as well as the genomes’ tissue distributions and prevalences in field ticks.

Mononegavirales and Rhabdoviridae

*Mononegavirales and Rhabdoviridae* sequences were the most commonly identified. We detected three new sequences of this family, each containing either four (Lobo virus) or five (Doud Peak virus) open reading frames (ORFs). Each of these viral genomes is similar to other rhabdoviruses identified in ticks, including two viruses identified in Australian ticks that were not previously assigned to a clade[^53^](https://www.zotero.org/google-docs/?UMUTCa)(Figure S8). These two viral sequences rarely occur in the same samples. Although this co-exclusion is not significant, it could indicate that these related viruses compete to occupy a similar niche in their host. Both were confirmed by PCR in salivary glands and in midguts, as well as in field-collected larvae and laboratory-reared adult females.

We assigned the North Fork virus to the *Mononegavirales*. It was identified in 15% of samples and mapped quite distinctly from Doud Peak virus and Lobos virus on the *Rhabdoviridae* RdRp phylogeny (Figure S8). Many of its closest relatives are other viruses identified in ticks. It harbors five ORFs, and its closest relative is an endogenous virus that was discovered in the *I. scapularis* cell line IDE8, which causes no apparent cytopathic effect, raising the possibility that North Fork virus may be a related endogenous virus of *I. pacificus*[^62^](https://www.zotero.org/google-docs/?doKBx9)*.* In keeping with this hypothesis, North Fork virus was identified in a pool of laboratory-reared *I. pacificus* larvae by PCR, suggesting that it is able to be transmitted directly to offspring (who have not yet been exposed to an animal host).

Bunyavirales

The Shoal Cavern virus L segment mapped to the *Phenuviridae* arm of the Bunyavirales RdRp phylogenetic tree (Figure S8). In addition to the RdRp-containing L segment, a putative S segment containing the nucleoprotein gene was identified by co-occurrence. The *Phenuviridae* family includes tick-borne viruses known to cause human disease such as severe fever with thrombocythemia virus and heartland virus. Shoal Cavern virus was not only highly prevalent (40%) but was also present in astonishingly high levels in some libraries, accounting for 38% of the total nonhost library in a single sample. This mirrors the previous finding of a phlebovirus discovered in the hard tick *Dermacentor occidentalis* in California[^15^](https://www.zotero.org/google-docs/?ZUGTmS), adding further evidence that such viruses may be common tick endosymbionts. Shoal Cavern virus was identified in both salivary gland and midgut tissue, and it may therefore be transmissible to bloodmeal hosts, although its presence in field larvae suggest it may also be vertically transmitted.

Two additional viral sequences were discovered that appear to group with other members of *Bunyavirales* but outside of known families (Figure S8). They are most closely related to viruses of the *Hantaviridae* family. We named these Soberanes virus and Painter’s Point virus. They each contain a single large, approximately 9-kilobase (kb) ORF. A glycoprotein-encoding genomic segment was identified for each of these viruses, as well as one (Painter’s Point virus) and two (Soberanes virus) ~1.7-kb genomic segments, similar in size to the canonical S segment, that however harbor ORFs of unknown function rather than the nucleoprotein gene. Soberanes virus has no evidence of vertical transmission, as it was identified only in wild collected adult ticks (Figure 3e). Painter’s Point virus was identified in field samples as well as laboratory adults, nymphs, and larvae, suggesting the ability to be vertically transmitted.

Chuviridae

We name a sequence belonging to *Chuviridae* Rocky Ridge virus. It is composed of a 10.8 kb genome with three ORFs. Since the discovery of this family, a number of chuviruses have been identified across continents and several tick families, and they have also frequently been found as endogenous viral elements in mosquito genomes[^83^](https://www.zotero.org/google-docs/?CHVOju). The polymerase of the chuvirus detected in our study shows >90% amino acid sequence identity with Suffolk virus, a virus identified in *I. scapularis*. Rocky Ridge virus was both highly prevalent (39%) and the most abundant virus of any in the dataset; nine samples contained more than 100,000 reads per million mapping to it. Rocky Ridge virus was identified in nearly all tick samples screened by PCR, and its presence in salivary glands suggests it may be transmissible to hosts, while its presence in larvae suggests it can also be vertically transmitted.

Narnaviridae

We identified a sequence most closely related to viruses from the *Narnaviridae* family and named it Portuguese Ridge virus (Figure S8). Narnaviruses are unique in that they lack any structural proteins or capsids, existing instead as ribonucleoprotein complexes which are transmitted directly cell-to-cell either vertically or sexually. While first identified as viruses of fungi, they have since been identified in a variety of arthropods, sometimes with additional segments or ambigrammatic open reading frames[^84^](https://www.zotero.org/google-docs/?7eIOuE). Portuguese Ridge virus was the only virus in the dataset to exhibit clear tissue tropism, identified only in midguts.

Reovirales

We identified a sequence with close homology to Coltiviruses within the *Reovirales* and named it Calla Lily Valley virus (Figure S8). Reoviruses have double stranded RNA genomes composed of up to 12 segments, and they infect a broad range of hosts including fungi, invertebrates, vertebrates, and plants. We identified an additional 8 segments by co-occurrence, four of which have homology to other *Reoviridae* proteins and four of which have no homology to known proteins (Figure 3a, Figure S5). Several members of this family have recently been identified in ticks and they are one of the most common families of endogenous tick viruses[^27,85,86^](https://www.zotero.org/google-docs/?KLRy3K) .

Solemoviridae

We identified a genome sequence with a polymerase gene that groups closely (>90% amino acid identity) with the polymerases of other viruses identified in ticks in the now defunct *Luteoviridae* family, and we named it Notley’s Landing virus (Figure S8). Despite the high read coverage of this genome, it is smaller than expected, with viruses of this family typically being 5-6 kb in length with six open reading frames. This could indicate that Notley’s Landing virus represents a new related family with a segmented genome, however no additional segments were identified by co-occurrence. Notley’s Landing virus was not detected by PCR in either midguts or salivary glands but it was detected in laboratory-reared larvae, indicating it could be vertically transmitted.

Picornaviridae

We identified a viral sequence that is a member of *Picornaviridae*, a family of monopartite ssRNA viruses of genome size 7-9 kb encoding a single polyprotein (Figure S8). We named it Cabrillo virus. This sequence is most closely related to Falcovirus A1, a virus identified in the common kestrel[^87^](https://www.zotero.org/google-docs/?StFMO8). It is possible that Cabrillo virus may be an avian-infecting virus as *I. pacificus* are known to feed on birds[^88^](https://www.zotero.org/google-docs/?LvQ1SS). It was identified in nearly all tick samples tested by PCR, indicating both horizontal and vertical transmission.

Ormycovirus

Two additional genomes containing an RdRp were found to have distant homology to members of the recently discovered ormycovirus clade[^89^](https://www.zotero.org/google-docs/?ZZ0qTH). Similar to narnaviruses, viruses of this clade lack structural proteins and likely exist as ribonucleoprotein complexes. While Kasler Point virus was identified by PCR in both field-collected and laboratory ticks (including larvae), Wildcat Canyon virus was only identified in field-collected ticks, indicating potentially different modes of transmission. Interestingly, several contigs that co-occurred with Kasler Point virus had homology to plant sequences, suggesting that it could be a plant virus that was sequenced on the exterior of the tick. Ticks or plants would both represent as yet unreported host tropism for this group of viruses, originally discovered in fungal sequencing data.

REFERENCES

83. Dezordi, F. Z., dos Santos Vasconcelos, C. R., Rezende, A. M. & Wallau, G. L. In and outs of Chuviridae endogenous viral elements: origin of a retrovirus and signature of ancient and ongoing arms race in mosquito genomes. <http://biorxiv.org/lookup/doi/10.1101/2020.02.15.950899> (2020) doi:10.1101/2020.02.15.950899.

84. DeRisi, J. L. et al. An exploration of ambigrammatic sequences in narnaviruses. Sci. Rep. 9, 17982 (2019).

85. Wille, M. et al. Sustained RNA virome diversity in Antarctic penguins and their ticks. ISME J. 14, 1768–1782 (2020).

86. Vanmechelen, B. et al. Exploration of the Ixodes ricinus virosphere unveils an extensive virus diversity including novel coltiviruses and other reoviruses. Virus Evol. 7, veab066 (2022).

87. Boros, Á. et al. Genome analysis of a novel, highly divergent picornavirus from common kestrel (Falco tinnunculus): The first non-enteroviral picornavirus with type-I-like IRES. Infect. Genet. Evol. 32, 425–431 (2015).

88. Eisen, L., Eisen, R. J. & Lane, R. S. The roles of birds, lizards, and rodents as hosts for the western black-legged tick Ixodes pacificus. J. Vector Ecol. J. Soc. Vector Ecol. 29, 295–308 (2004).

89. Forgia, M. et al. Three new clades of putative viral RNA-dependent RNA polymerases with rare or unique catalytic triads discovered in libraries of ORFans from powdery mildews and the yeast of oenological interest Starmerella bacillaris. Virus Evol. 8, veac038 (2022).
