# Supplementary material for: Metatranscriptomic investigation of single Ixodes pacificus ticks reveals diverse microbes, viruses, and novel mRNA-like endogenous viral elements
Source: mSystems. 2024 May 14;9(6):e00321-24. doi: 10.1128/msystems.00321-24 (PMC11237458; doi:10.1128/msystems.00321-24)
Supplement: Table S1 — Summary of the viruses identified. [file msystems.00321-24-s0003.docx]

Table S1. Summary Information for Assembled Viral Genome Sequences

| Virus Name* | Provisional taxonomic assignment** | Prevalence | Length (bp)*** | Number of detected ORFs | Best RdRp blastp match**** | % Amino acid identity |
| --- | --- | --- | --- | --- | --- | --- |
| Shoal Cavern Virus | *Phenuiviridae* | 0.40 | 6857 | 1 | AII01801.1 L protein [Blacklegged tick phlebovirus 1] | 78.5 |
| Rocky Ridge Virus | *Chuviridae* | 0.39 | 10808 | 3 | YP_009177218.1 polymerase [Suffolk virus] | 91.3 |
| Lobos Virus | *Rhabdoviridae* | 0.37 | 10509 | 4 | ASY03266.1 RNA-dependent RNA-polymerase [Norway mononegavirus 1] | 47.9 |
| Soberanes Virus | *Bunyavirales* | 0.19 | 9414 | 2 | QKK82912.1 RNA-dependent RNA polymerase [Ubmeje virus] | 43.8 |
| North Fork Virus | *Mononegavirales* | 0.15 | 11875 | 5 | QJQ27116.1 putative RdRp protein [IRE/CTVM19-associated rhabdovirus] | 74.3 |
| Doud Peak Virus | *Rhabdoviridae* | 0.11 | 11738 | 6 | ASY03261.1 RNA-dependent RNA-polymerase [Norway mononegavirus 1] | 76.4 |
| Notleys Landing Virus | *Solemoviridae* | 0.10 | 2711 | 2 | BBD75429.1 hypothetical protein, partial [Ixodes scapularis associated virus 1] | 93.6 |
| Cabrillo Virus | *Picornaviridae* | 0.07 | 8183 | 1 | YP_009133208.1 polyprotein [Falcovirus A1] | 49.3 |
| Kasler Point Virus | Unknown | 0.05 | 3182 | 1 | None | Not applicable |
| Painters Point Virus | *Bunyavirales* | 0.04 | 9159 | 2 | ASY03250.1 RNA-dependent RNA-polymerase [Bronnoya virus] | 69.1 |
| Calla Lily Valley Virus | *Reoviridae* | 0.02 | 4300 | 1 | QKK82923.1 VP1 [Fennes virus] | 56.5 |
| Portuguese Ridge Virus | *Narnaviridae* | 0.02 | 2593 | 1 | QBC65280.1 RNA-dependent RNA polymerase, partial [Rhizopus microsporus 20S narnavirus] | 36.5 |
| Wildcat Canyon Virus | Unknown | 0.02 | 1767 | 1 | None | Not applicable |

*Based on the geographical features of the region from which the tick samples harboring the viral genomes were collected. **Based on phylogenetic analysis of RdRp amino acid sequence (Methods, Figure S9). ***For multi-segmented viruses (Shoal Cavern Virus, Soberanes Virus, Painter’s Point Virus, and Calla Lilly Valley Virus) only RdRp gene segment length is shown. ****Queried against the full NCBI nonredundant protein database (as of January 24, 2021) using diamond blastp version 0.9.24[^73^](https://www.zotero.org/google-docs/?lX2oU8). For RdRps with multiple hits, the alignment with the highest bitscore is reported.
