# Supplementary material for: Metatranscriptomic investigation of single Ixodes pacificus ticks reveals diverse microbes, viruses, and novel mRNA-like endogenous viral elements
Source: mSystems. 2024 May 14;9(6):e00321-24. doi: 10.1128/msystems.00321-24 (PMC11237458; doi:10.1128/msystems.00321-24)
Supplement: Table S2 — All PCR primer sequences used. [file msystems.00321-24-s0004.docx]

| **Target**  **Sequence**  **Name** | **Primer**  **Name** | **Primer Sequence** | **Expected**  **Band Size** |
| --- | --- | --- | --- |
| vlt111.1 | vlt111_F.1 | CTGTGCAGTTCCGATGTGGA | 1074 |
| vlt111.1 | vlt111_R.1 | CCACTGTTCCCGAGGTTTGT | 1074 |
| vlt111.2 | vlt111_F.2 | TTTCTCTCAACGCTCGCAGT | 1010 |
| vlt111.2 | vlt111_R.2 | CTTGAACGTCCGAGCTCACT | 1010 |
| vlt111.3 | vlt111_F.3 | CCTGTGGTACGGGATCCAAG | 1038 |
| vlt111.3 | vlt111_R.3 | TCAACCATTGCCAGAAACGC | 1038 |
| vlt111.4 | vlt111_F.4 | CTGGCGCTCTGTTTCTTTGG | 1025 |
| vlt111.4 | vlt111_R.5 | AGCTCGAGAAAGAGGACCTT | 1025 |
| vlt111.5 | vlt111_F.5 | TCCTTGCCCGAGTGAATTGT | 561 |
| vlt111.5 | vlt111_R.5 | GTTCTTCACGCAACGCCAAT | 561 |
| vlt111.6 | vlt111_F.6 | CAGCAGGGTCGATTGTCTGT | 839 |
| vlt111.6 | vlt111_R.6 | TTAGTGCGTAACGAGCTGGG | 839 |
| vlt111.7 | vlt111_F.7 | CCGGGACCACTACGATTTGT | 554 |
| vlt111.7 | vlt111_R.7 | TGATGAAAGACCTCAGGCCG | 554 |
| vlt111.8 | vlt111_F.8 | CCCAGCTATGGAACCCTTCG | 640 |
| vlt111.8 | vlt111_R.8 | CAGGGTACAGCTCTGACGAG | 640 |
| vlt_307 | vlt_307_F | CGCCTAGGGTCGATCAACAA | 800 |
| vlt_307 | vlt_307_R | TTTTGGCGATTCCGTCCTCA | 800 |
| vlt_246 | vlt_246_F | TTGGCGATCTCCCTCAGAAC | 225 |
| vlt_246 | vlt_246_R | AGGAACGGCAGGCTAATTCC | 225 |
| vlt_370 | vlt_370_F | GTCGGCATGCGTGGTAAATC | 488 |
| vlt_370 | vlt_370_R | ACGTCATGACCTGACCAACC | 488 |
| vlt_481 | vlt_481_F | AAGTGCCAGGACGAGAACTG | 1049 |
| vlt_481 | vlt_481_R | CACGTTTCTCACCTTCGGGA | 1049 |
| vlt_2629 | vlt_2629_F | GCTCTAGCTACCCGACGTTC | 867 |
| vlt_2629 | vlt_2629_R | ATTGCTCTGGGCCAGTAACC | 867 |
| vlt_3893 | vlt_3893_F | CTTCGAACCCGTCCGAGAAA | 686 |
| vlt_3893 | vlt_3893_R | ATTGGTTCCTTCTGCCCGAG | 686 |
| vlt_3215 | vlt_3215_F | GTGAGGAGCGATTGGTTGTTTAAAGC | 372 |
| vlt_3215 | vlt_3215_R | TGACTCCATCATCAATCACATACTTGTCAG | 372 |
| vlt_246 | vlt_246_F | AGATACTGGCTGACCTGTGGC | 247 |
| vlt_246 | vlt_246_R | GTCTTCTCTTTCTTTGCTGTCGTGTTG | 247 |
| vlt_41 | vlt_41_F | TTCTGATGGTCTTGGCATTCTGGAAG | 400 |
| vlt_41 | vlt_41_R | CTGTCGTTCTGGAAGGCGGTTC | 400 |
| Calla Lily Valley Virus | clvv_F | TGTGGTACAGGAACTTTATCACGATCAC | 255 |
| Calla Lily Valley Virus | clvv_R | CGGGTGAGGTTTAGGATACTCAATGG | 255 |
| Cabrillo Virus | cv_F | CATCAACAACATCACTCTGTTCCTCCAG | 141 |
| Cabrillo Virus | cv_R | TGACATACTCATATGCTTGCACTCTGTC | 141 |
| Doud Peak Virus | dpv_F | CGAGCATGCCTTTCTGTGG | 111 |
| Doud Peak Virus | dpv_R | GCACGGAGGAGACTTGGATAGC | 111 |
| Notley's Landing Virus | nlv_F | AGTCGCTCCGCTTTCCCATCA | 423 |
| Notley's Landing Virus | nlv_R | CCTTATTCCGTTCTTGCTCTACATCGT | 423 |
| Panters Point Virus | ppv_R | GACAGTCACGGTGTAGTCAATTATGGT | 353 |
| Panters Point Virus | ppv_R | TGGAGTCCTTCCTGGCAAGCAT | 353 |
| Rocky Ridge Virus | rrv_F | AACTCGGCTTCTGCTCTTCTACCT | 204 |
| Rocky Ridge Virus | rrv_R | TGTATGATGCTTCTGGCTCTGTCTTG | 204 |
| Lobos Virus | lv_F | AATAACACCAACCAACACCAAGCAATC | 161 |
| Lobos Virus | lv_R | GCAACATCATCATCTTCCATCGGTAGTA | 161 |
| Wildcat Canyon Virus | wcv_F | CCAAAGGAGACTGGGTATCTGTATTCATC | 489 |
| Wildcat Canyon Virus | wcv_R | GCCTATAAGAGGTCACGGACAAGGTA | 489 |
| North Fork Virus | nfv_F | GATGAGGCTGGATGGCTGATTGTC | 371 |
| North Fork Virus | nfv_R | CACCCTTGATGAGATTTCCCGTCTTT | 371 |
| Soberanes Virus | sv_F | TATTGAGTGACAGCGGTGGCATTG | 148 |
| Soberanes Virus | sv_R | GAGAACGGTCTGGCACAGTAGGTA | 148 |
| Kasler Point Virus | kpv_F | CTCGGAGAAGTCTTAGGAATGGAAGTTC | 101 |
| Kasler Point Virus | kpv_R | CTTAGCCATGCTTAGGATACCGTTCAA | 101 |
| Shoal Cavern Virus | scv_F | ACCATTCTCCACACCTCCTCCATC | 548 |
| Shoal Cavern Virus | scv_R | GCAGTCTCAGGCAACAAGAACAGT | 548 |
| Portuguese Ridge Virus | prv_F | TTCCACCGAAGACCTTGAGTCACT | 261 |
| Portuguese Ridge Virus | prv_R | GCGACCTCAGCAGAGACCATCT | 261 |

**Table S2. List of all PCR primer sequences used**
